# Synthesis, cytotoxicities, and carbonic anhydrase inhibition potential of 6-(3-aryl-2-propenoyl)-2(*3H*)-benzoxazolones

**DOI:** 10.1080/14756366.2019.1670657

**Published:** 2019-10-02

**Authors:** Sinan Bilginer, Halise Inci Gul, Feyza Sena Erdal, Hiroshi Sakagami, Serkan Levent, Ilhami Gulcin, Claudiu T. Supuran

**Affiliations:** a Department of Pharmaceutical Chemistry, Faculty of Pharmacy, Ataturk University, Erzurum, Turkey;; b School of Dentistry, Meikai University Research Institute of Odontology (M-RIO), Meikai University, Sakado, Japan;; c Department of Pharmaceutical Chemistry, Faculty of Pharmacy, Anadolu University, Eskisehir, Turkey;; d Department of Chemistry, Faculty of Science, Ataturk University, Erzurum, Turkey;; e Neurofarba Department, University Firenze, Florence, Italy

**Keywords:** Anticancer, benzoxazolone, carbonic anhydrase, chalcone, cytotoxic

## Abstract

In this study, new chalcone compounds having the chemical structure of 6-(3-aryl-2-propenoyl)-2(*3H*)-benzoxazolones (**1–8**) were synthesised and were characterised by ^1^H-NMR, ^13 ^C-NMR, and HRMS spectra. Cytotoxic and carbonic anhydrase (CA) inhibitory effects of the compounds were investigated. Cytotoxicity results pointed out that compound **4**, 6-[3-(4-trifluoromethylphenyl)-2-propenoyl]-3*H*-benzoxazol-2-one, showed the highest cytotoxicity (CC_50_) and potency-selectivity expression (PSE) value, and thus can be considered as a lead compound of this study. According to the CA inhibitory results, IC_50_ values of the compounds **1–8** towards hCA I were in the range of 29.74–69.57 µM, while they were in the range of 18.14 – 48.46 µM towards hCA II isoenzyme. K*_i_* values of the compounds **1–8** towards hCA I were in the range of 28.37 ± 6.63–70.58 ± 6.67 µM towards hCA I isoenzyme and they were in the range of 10.85 ± 2.14 – 37.96 ± 2.36 µM towards hCA II isoenzyme.

## Introduction

Cancer is the second cause of death in the world after the cardiovascular system diseases. According to The World Health Organisation (WHO) report, 13.1 million people will die because of cancer by 2030^1^. Anticancer drugs in clinics have several adverse effects such as nausea, vomiting, hair loss, and pain, in addition to low selectivity and drug resistance problems^2^. Their associated limitations and adverse effects are still prompting the researchers to develop more potent, selective, and safer anticancer drug candidates. Chemotherapeutic drugs commonly used for cancer treatment in clinics are alkylating anticancer agents. These compounds interact with amino and hydroxyl groups which are available nucleic acids and proteins and lead to unwanted side effects in other tissues except neoplasms^3^.

α,β-unsaturated ketones are bioactive moieties having alkylation ability. They have an affinity for thiols^4^^,^^5^ while they are either inert or far less reactive towards amino and hydroxyl groups which are available nucleic acids^6^^,^^7^. It was reported that before the cell division, level of glutathione, which is a thiol compound, increases^8^. That is why it can be supposed that compounds which are thiol selective alkylators may perform selective toxicity against tumour tissues^9–11^ and these types of compounds may have advantages over available anticancer drugs in the market.

Chalcones are 1,3-diaryl-2-propen-1-ones, which consist of two aromatic rings connected by a three-carbons chain including α,β-unsaturated carbonyl system^12^. Depending on substitution on the aryl ring, chalcones have a wide range of biological activities such as antiinflammatory^13^^,^^14^, antimicrobial^15^^,^^16^, antioxidant^17^, cytotoxic/anticancer^18^^,^^19^, chemopreventive^20^, topoisomerase inhibiting^4^, carbonic anhydrase (CA) inhibiting^21^^,^^22^ and acetylcholine esterase inhibiting activities^23^.

Benzoxazolones are considered as “privileged scaffolds” in the design of pharmacological probes^24^. Benzoxazolones have high flexibility for chemical modifications allowing changes to the characteristics of side-chains on a rigid platform^25^. As a result, benzoxazolones exhibit various biological activities such as anti-HIV^26^, anticancer^27^^,^^28^, analgesic^29^, antiinflammatory^30^, antimicrobial^31^, and antioxidant^32^ activities. The functionalisation of the nitrogen atom at the third position of the benzoxazolone moiety is of interest since the electronic characteristics of this atom can be decisive for the biological activity^24^.

Some chalcones bearing 2(3H)-benzoxazolone were reported with strong cytotoxic activity^33^^,^^34^. However, there is a very limited number of studies about them. One of them is Ivanova and co-workers’ study. They reported antileukemic effects of benzoxazolone derived chalcones on BV-173 cell-line by inducing apoptotic cell death^33^. The fact that these compounds showed selective and strong anticancer activity pointed out the importance of benzoxazolone bearing chalcone molecules in designing new anticancer candidate molecules.

CA is a metalloenzyme that catalyses the interconversion between CO_2_ and bicarbonate. CAs play an important role in many physiological and pathological processes such as biosynthetic reactions (such as gluconeogenesis, lipogenesis, and ureagenesis), respiration and transport of CO_2_/bicarbonate, electrolyte secretion in a variety of tissues/organs, calcification, and tumorigenicity^35^^,^^36^.

There are eight CA families which are genetically different such as the α-, β-, γ-, δ-, ζ- η-, θ-, and the recently reported ι-CAs^37^. α-CAs are available in human and it has 15 subtypes. Three of them (CA VIII, X, and XI) are non-catalytic as do not possess a zinc ion in their active site. The other 12 CA isozymes have differences in terms of catalytic activity, location, and cell distributions. According to the location, these are cytosolic isozymes (CA I, II, III, VII, and XIII), membrane-bounded isozymes (CA IV, IX, XII, and XIV), secreted salivary isozyme (CA VI), and mitochondrial isozyme (CA V)^38–40^.

Activation or inhibition of several CAs is a potential strategy for diagnosis and/or treatment of several diseases such as glaucoma, epilepsy, several neurological diseases, and obesity. Antiglaucomal agents (CA II, IV, and XII), diuretics (CA II, IV, XII, and XIV), antiepileptics (CA VII, and XIV) inhibit several isoenzymes of CA. Some chalcone compounds have been reported as attractive scaffolds for the development of new CA inhibitors^21^^,^^41^.

CA inhibitors commonly include sulfonamide^35^^,^^42^ or phenol^22^^,^^35^^,^^41^ functional group. However, there are several studies reporting that chalcone compounds without these functional groups showed potent CA inhibiting effect^43–46^. In addition, there was no study about CA inhibiting potency of benzoxazolone or its chalcone derivatives.

In this study, it was aimed to synthesise some chalcone compounds having the chemical structure of 6-(3-aryl-2-propenoyl)-2(*3H*)-benzoxazolones (**1–8**) in which α,β-unsaturated ketone moiety is available to evaluate their cytotoxicity (towards both tumour cell lines and non-tumour cells) and inhibition profile towards CA hCAI and II with the expectation to find out a new candidate molecule/s which may direct further designs.

## Experimental

### Chemistry

The nuclear magnetic resonance (NMR) spectra (^1^H-NMR, and ^13^C-NMR) were recorded on a Bruker AVANCE III 400 MHz (Bruker, Karlsruhe, Germany) spectrometer [400 MHz (^1^H) and 100 MHz (^13^C)]. Chemical shifts are given as δ values in ppm against tetramethylsilane as the internal standard and *J* values were expressed in Hz. Mass spectra of the compounds were taken using a liquid chromatography ion trap-time of flight tandem mass spectrometer (Shimadzu, Kyoto, Japan) equipped with an electrospray ionisation (ESI) source, operating in both positive and negative ionisation mode. Shimadzu’s LCMS Solution software was used for data analysis. Melting points were determined using an Electrothermal 9100 instrument (IA9100, Bibby Scientific Limited, Staffordshire, UK) and are uncorrected. Reactions were monitored by thin-layer chromatography (TLC) using silica gel 60 HF254 (Merck KGaA, Darmstadt, Germany).

### Synthesis of 6-acetyl-2(3H)-benzoxazolone

**Scheme 1.**
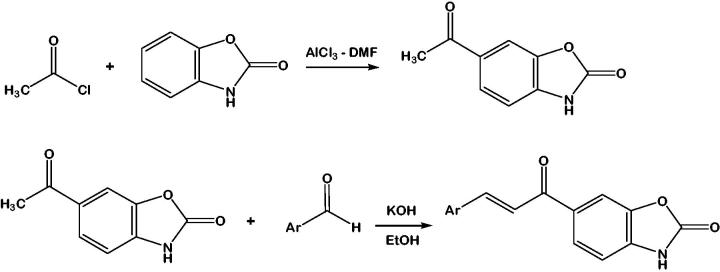
Synthesis of the compounds **1–8**. **Ar:** Phenyl (**1**); 4-methylphenyl (**2**); 4-Methoxyphenyl (**3**); 4-trifluoromethylphenyl (**4**); 3-hyrdoxyphenyl (**5**); 4-isopropylphenyl (**6**); 4-dimethylaminophenyl (**7**); 4-benzyloxyphenyl (**8**).

Dimethylformamide (13 ml, 172 mmol) was slowly added on aluminum chloride (80 g, 600 mmol). The mixture was heated at 45 °C for 5 min. 2(*3H*)-benzoxazolone (8.1 g, 60 mmol) and acetyl chloride (6,4 ml, 90 mmol) were added into the solution of aluminum chloride (Scheme 1). Then the reaction mixture was heated at 80 °C for 3 h and poured on ice water (200 ml) with HCl (30 ml, 37%). The precipitated crude product was collected by filtration and it was air-dried and crystallised from ethanol. (Yield: 77%, m.p: 231–234 °C, brown crystals)^27^.

### Synthesis of chalcone compounds 1, 2, 3, 4, and 6

To the mixture of 6-acetyl-2(*3H*)-benzoxazolone (ketone, 5.6 mmol) and a suitable aldehyde [benzahdehyde (**1**), 4-methylbenzaldehyde (**2**), 4-methoxybenzaldehyde (**3**), 4-trifluorobenzaldehyde (**4**), 4-isopropylbenzaldehyde (**6**)] in ethanol (5 ml) in 1:1 mol ratios, aqueous solution of KOH (10%, 5 ml) was added (Scheme 1). Reaction content was stirred at room temperature for 24 h. Reactions were followed by TLC. When the reaction finished, the content of the reaction flask was poured on ice-water (100 ml) and neutralised by HCl (37%). The precipitated solid product was filtered and washed with cold water^27^. The crude compounds were purified by crystallisation from a suitable solvent (Acetonitrile: ethanol for compounds **1–4**; acetonitrile: methanol for compound **6**)^41^.

#### 6-(3-Phenyl-2-propenoyl)-3H-benzoxazol-2-one (1)

Yield 77%. Mp: 230–232 °C. 1H-NMR (DMSO-d_6_) δ (ppm) 12.03(bs, 1H, NH), 8.11(d, 1H, arom. H, *J* = 1.2 Hz), 8.07(dd,1H, arom. H, *J*_1_*_ _*= 8.2 Hz, *J*_2_*_ _*= 1.6 Hz), 7.99(d,1H, Ar-CH=, *J* = 15.5 Hz), 7.89–7.91(m, 2H, arom. H), 7.74(d, 1H, =CHCO, *J* = 15.5 Hz), 7.47(d, 1H, arom. H, *J* = 1.2 Hz), 7.45(d, 2H, arom. H, *J* = 2.5 Hz), 7.24(d, 1H, arom. H, *J* = 8.2 Hz). ^13^C-NMR (DMSO-d_6_) δ (ppm) 187.8, 154.9, 144.2, 143.9, 135.5, 135.2, 132.3, 131.0, 129.38, 129.37, 129.36, 126.2, 122.3, 110.0. HRMS (ESI-MS) *m/z* calculated [M + H]^+^ 266.0812; measured 266.0803.

#### 6-[3-(4-Metyhlphenyl)-2-propenoyl]-3H-benzoxazol-2-one (2)

Yield 83%. Mp: 258–260 °C. 1H-NMR (DMSO-d_6_) δ (ppm) 8.08 (d, 1H, arom. H, *J* = 1.5 Hz), 8.05 (dd,1H, arom. H, *J*_1_= 8.1 Hz, *J*_2_ = 1.5 Hz), 7.93 (d, 1H, Ar-CH=, *J* = 15.5 Hz), 7.79 (d, 2H, arom. H, *J* = 8.0 Hz), 7.70 (d, 1H, =CHCO, *J* = 15.5 Hz), 7.27 (d, 2H, arom. H, *J* = 8.0 Hz), 7.22 (d, 1H, arom. H, *J* = 8.1 Hz), 2.35 (s, 3H, CH_3_). ^13^C-NMR (DMSO-d_6_) δ (ppm) 187.7, 155.2, 144.2, 144.0, 141.1, 135.9, 132.5, 132.3, 129.98, 129.97, 129.42, 129.41, 126.1, 121.2, 110.0, 109.8, 21,6. HRMS (ESI-MS) *m/z* calculated [M + H]^+^ 280.0968; measured 280.0967.

#### 6-[3-(4-Methoxyphenyl)-2-propenoyl]-3H-benzoxazol-2-one (3)

Yield 58%. Mp: 210–213 °C. 1H-NMR (DMSO-d_6_) δ (ppm) 8.08 (d, 1H, arom. H, *J* = 1.4 Hz), 8.04 (dd, 1H, arom. H, *J*_1_*_ _*= 8.2 Hz, *J*_2_*_ _*=1.4 Hz), 7.86 (d, 2H, arom. H, *J* = 8.8 Hz), 7.84 (d, 1H, Ar-CH =, *J* = 15.5 Hz), 7.71 (d, 1H, =CHCO, *J* = 15.5 Hz), 7.22 (d,1H, arom. H, *J* = 8.2 Hz), 7.01 (d,2H, arom. H, *J* = 8.8 Hz), 3.82 (s, 3H, =OCH_3_). ^13^C-NMR (DMSO-d_6_) δ (ppm) 187.6, 161.8, 155.0, 144.2, 143.9, 135.3, 132.6, 131.3, 127.9, 126.0, 119.7, 114.8, 110.0, 109.9, 55.9. HRMS (ESI-MS) *m/z* calculated [M + H]^+^ 296.0917; measured 296.0918.

#### 6-[3-(4-Trifluoromethylphenyl)-2-propenoyl]-3H-benzoxazol-2-one (4)

Yield 80%. Mp: 257–259 °C. 1H-NMR (DMSO-d_6_) δ (ppm) 7.24 (d, 1H, arom. H, *J* = 8.2 Hz), 7.78 (d,1H, =CHCO, *J* = 15.7 Hz), 7.80 (d, 2H, arom. H, *J* = 8.2 Hz), 8.06 (d, 1H, arom. H, *J* = 1.6 Hz), 8.09 (d, 1H, Ar-CH =, *J* = 15.7 Hz), 8.12–8.14 (m, 3H, arom. H). ^13^C-NMR (DMSO-d_6_) δ (ppm) 187.7, 154.9, 143.9, 142.2, 139.2, 135.7, 131.9, 130.5, 129.9, 126.2, 124.9, 122.7, 119.1, 110.1. HRMS (ESI-MS) *m/z* calculated [M + H]^+^ 334.0686; measured 334.0687.

#### 6-[3-(4-Isopropylphenyl)-2-propenoyl]-3H-benzoxazol-2-one (6)

Yield 33%. Mp: 220–222 °C. 1H-NMR (DMSO-d_6_) δ (ppm) 12.08 (bs, 1H, NH), 8.10 (d, 1H, arom. H, *J* = 1.4 Hz), 8.06 (dd, 1H, arom. H, *J*_1_=8.2 Hz, *J*_2_*_ _*=1.4 Hz), 7.93 (d, 1H, Ar-CH=, *J* = 15.5 Hz), 7.82 (d, 2H, arom. H, *J* = 8.2 Hz), 7.72 (1H, d, =CHCO, *J* = 15.5 Hz) 7.34 (d, 2H, arom. H, *J* = 8.2 Hz), 7.24 (d, 1H, arom. H, *J* = 8.2 Hz), 2.98–2.89 (m, 1H,CH), 1.22 (d, 6H, CH_3_, *J* = 6.9 Hz). ^13^C-NMR (DMSO-d_6_) δ (ppm) 187.8, 154.9, 151.9, 144.3, 143.9, 135.3, 132.9, 132.4, 129.55, 129.54, 127.35, 126.12, 121.3, 110.0, 33.9, 24.1. HRMS (ESI-MS) *m/z* calculated [M + H]^+^ 308.1281; measured 308.1286.

### Synthesis of the compounds 5, 7, and 8

To the mixture of 6-acetyl-2(*3H*)-benzoxazolone (ketone, 5.6 mmol) and a suitable aldehyde [3-hydroxybenzaldehyde (**5**), 4-dimethylaminobenzaldehyde (**7**), 4-benzyloxybenzaldehyde (**8**)] in 1:1 mol ratios in ethanol (2 ml), aqueous solution of KOH (10%, 2 ml) was added. Then the mixture was irradiated by microwave (50–80 °C, 25–60 W) for 15 min (compound **7**), 20 min (compound **8**) and 30 min (compound **5**). Reactions were followed by TLC. When the reaction finished, the content of the reaction flask was poured on ice-water (100 ml) and neutralised by HCl (37%). The solid precipitated was filtered and washed with cold water. The crude compounds were purified by crystallisation from acetonitrile.

The chemical structures of the compounds were confirmed by ^1^H-NMR, ^13^C-NMR, and HRMS.

#### 6-[3-(3-Hydroxyphenyl)-2-propenoyl]-3H-benzoxazol-2-one (5)

Yield 44%. Mp: 277–279 °C. 1H-NMR (DMSO-d_6_) δ (ppm) 12.10 (bs, 1H, NH), 9.61(s, 1H, OH), 8.08 (d, 1H, arom. H, *J* = 1.5 Hz), 8.03 (dd, 1H, arom. H, *J*_1_*_ _*= 8.4 Hz, *J*_2_*_ _*=1.5 Hz), 7.86 (d, 1H, Ar-CH =, *J* = 15.4 Hz), 7.62 (d, 1H, =CHCO, *J* = 15.4 Hz), 7.31 (d, 1H, arom. H, *J* = 7.7 Hz), 7.24 (d, 1H, arom. H, *J* = 7.7 Hz), 7.21 (d, 2H, arom. H, *J* = 8.05 Hz), 6.85 (dd, 1H, arom. H, *J*_1_*_ _*= 8.05 Hz, *J*_2_*_ _*=1.5 Hz). ^13^C-NMR (DMSO-d_6_) δ (ppm) 188.1, 158.4, 155.2, 144.7, 144.2, 136.7, 135.6, 132.6, 130.5, 126.4, 120.6, 118.4, 116.0, 110.2. HRMS (ESI-MS) *m/z* calculated [M + H]^+^ 282.0761; measured 282.0746.

#### 6-[3-(4-Dimethylaminophenyl)-2-propenoyl]-3H-benzoxazol-2-one (7)

Yield 48%. Mp: 248–250 °C. 1H-NMR (DMSO-d_6_) δ (ppm) 12.02 (1 H, bs, NH), 8.00 (d,1H, arom. H, *J* = 8.3 Hz), 7.85 (d, 1H, Ar-CH =, *J* = 10.4 Hz), 7.70 (d,2H, arom. H, *J* = 8.9 Hz), 7.67 (d, 1H, =CHCO, *J* = 10.4 Hz), 7.66 (s,1H, arom. H), 7.19 (d, 1H, arom. H, *J* = 8.3 Hz), 6.73 (d, 2H, arom. H, *J* = 8.9 Hz), 2.99 (s, 6H, CH_3_). ^13 ^C-NMR (DMSO-d_6_) δ (ppm) 187.0, 155.2, 152.7, 145.7, 144.1, 135.1, 133.3, 131.5, 125.9, 122.8, 116.4, 112.4, 110.1, 110.0, 40.6. HRMS (ESI-MS) *m/z* calculated [M + H]^+^ 309.1234; measured 309.1230.

#### 6-[3-(4-Benzyloxyphenyl)-2-propenoyl]-3H-benzoxazol-2-one (8)

Yield 63%. Mp: 243–245 °C. 1H-NMR (DMSO-d_6_) δ (ppm) 8.07 (s, 1H, arom. H), 8.03 (dd, 1H, arom. H, *J*_1_*_ _*= 8.2 Hz, *J*_2_*_ _*= 1.3 Hz), 7.87–7.82 (m, 3H, arom. H), 7.69 (d, 1H, arom. H, *J* = 15.4 Hz), 7.46–7.30 (m, 4H, arom. H), 7.20 (d, 2H, arom. H, *J* = 8.4 Hz), 7.08 (d, 2H, arom. H, *J* = 8.8 Hz), 5.20 (s, 2H, CH_2_). ^13^C-NMR (DMSO-d_6_) δ (ppm) 187.8, 161.1, 155.3, 144.4, 144.2, 137.4, 135.6, 132.7, 131.5, 129.2, 128.6, 128.5, 128.3, 126.2, 120.0, 115.9, 110.2, 110.1, 70.1. HRMS (ESI-MS) *m/z* calculated [M + H]^+^ 372.1230; measured 372.1218.

## Biological activity

### Cytotoxicity test

#### Materials

The following chemicals and reagents were obtained from the indicated companies: Dulbecco’s modified Eagle’s medium (DMEM) from GIBCO BRL (Grand Island, NY); foetal bovine serum (FBS), 3-(4,5-dimethylthiazol-2-yl)-2,5-diphenyltetrazolium bromide (MTT), doxorubicin (DXR), and dimethyl sulphoxide (DMSO) from Wako Pure Chem. Ind. (Osaka, Japan); and culture plastic dishes and plates (96-well) were purchased from Becton Dickinson (Franklin Lakes, NJ).

#### Cell culture

Human normal oral mesenchymal cells, gingival fibroblast (HGF), and periodontal ligament fibroblast (HPLF) established from the first premolar tooth extracted from the lower jaw of a 12-year-old girl^47^ and human OSCC cell line HSC-2 (derived from tongue), purchased from Riken Cell Bank (Tsukuba, Japan), were cultured at 37 °C in DMEM supplemented with 10% heat-inactivated FBS, 100 units/ml penicillin G, and 100 µg/ml streptomycin sulphate under a humidified 5% CO_2_ atmosphere. HGF and HPLF cells at 10–18 population doubling levels were used in this study.

#### Assay for cytotoxic activity

Cells were inoculated at 2.5 × 10^3^ cells/0.1 ml in a 96-microwell plate (Becton Dickinson Labware, Franklin Lakes, NJ). After 48 h, the medium was replaced with 0.1 ml of fresh medium containing different concentrations of single test compounds. Cells were incubated further for 48 h and the relative viable cell number was then determined by the MTT method^22^^,^^48–54^. All benzoxazolone derivatives were dissolved with DMSO at the concentration of 40 mM and stored until use. Control cells were treated with the same amounts of DMSO (0.00156, 0.03125, 0.0625, 0.125, 0.25, 0.5, and 1.0%) and the cell damage induced by DMSO was subtracted from that induced by test agents. In brief, cells were stained with MTT reagent, dissolved with DMSO, and the absorbance of the MTT-stained cell lysate was measured at 560 nm, using a microplate reader (Infinite F 50R, TECAN, Kawasaki, Japan). Control cells were treated with the same amounts of DMSO and the cell damage induced by DMSO was subtracted from that induced by test agents. The concentration of compound that reduced the viable cell number by 50% (CC_50_) was determined from the dose-response curve and the mean value of CC_50_ for each cell type was calculated from triplicate assays.

#### Calculation of tumour specificity

Tumour specificity (TS) was calculated using the following equation: TS = Mean CC_50_ against three normal oral cell types (HGF, HPLF)/Mean CC_50_ against four OSCC cell lines (HSC-2). Since HGF cells were derived from gingival tissue, the relative sensitivity of these cells was also compared (as mean CC_50_ against HGF/mean CC_50_ against HSC-2).

#### Calculation of potency-selectivity expression

Potency-selectivity expression (PSE) was calculated by the following equation: PSE = Mean CC_50_ against two normal oral cell types/(CC_50_ against four OSCC cell lines)^2^×100 (HGF, HPLF, HSC-2) and as mean CC_50_ against HGF/(CC_50_ against HSC-2)^2^×100 using the pair of cell types from the same tissue (gingiva).

### Carbonic anhydrase inhibition

The purification of cytosolic CA isoenzymes (CA I and CA II) was previously described with a simple one-step method by a Sepharose-4B-L tyrosine-sulphanilamide affinity chromatography^42^^,^^53^^,^^55–58^. The protein quantity in the column effluents was determined spectrophotometrically at 280 nm. Sodium dodecyl sulphate-polyacrylamide gel electrophoresis (SDS-PAGE) was applied with a Bio-Rad Mini Gel system Mini-PROTEIN system (Bio-Rad Laboratories, Inc., Shanghai, China) after purification of both CA isoenzymes. Briefly, it was performed in acrylamide for the running (10%) and the stacking gel (3%) contained SDS (0.1%), respectively. The increase in absorbance of the reaction medium was spectrophotometrically recorded at 348 nm. Also, the quantity of protein was determined at 595 nm according to the method as described previously^55–60^. Bovine serum albumin was used as a standard protein. The IC_50_ values were obtained from activity (%) *versus* compounds plots. For the calculation of K*_i_* values, three different concentrations were used. The Lineweaver–Burk curves were drawn and calculations were realised as before^55–60^.

## Results and discussion

### Chemistry

The compounds **1–8**, 6-(3-aryl-2-propenoyl)-2(*3H*)-benzoxazolones, were synthesised successfully according to Scheme 1. Aryl part was changed as phenyl (**1**), 4-methylphenyl (**2**), 4-methoxyphenyl (**3**), 4-trifluoromethylphenyl (**4**), 3-hydroxyphenyl (**5**), 4-isopropylphenyl (**6**), 4-dimethylaminophenyl (**7**), and 4-benzyloxyphenyl (**8**). The compounds except **1, 3, 5** were reported for the first time in this study.

As indicated in Scheme 1, 6-acetyl-2(*3H*)-benzoxazolone was synthesised by Friedel–Crafts acylation first. The product was obtained in good yield and purity. Since direct acylation of 2(*3H*)-benzoxazolone is regioselective and always leads to a 6-acyl derivative^34^.

The chalcones **1–4**, and **6** were synthesised by the conventional synthesis method using Claisen–Schmidt condensation reaction between 6-acetyl-2(*3H*)-benzoxazolone and suitable aldehyde as shown in Scheme 1. On the other hand, chalcones **5, 6, 7,** and **8** were synthesised by microwave irradiation method.

The structures of the compounds were confirmed by ^1^H-NMR, ^13^C-NMR, and HRMS. In particular, analysis of ^1^H-NMR spectra of the compounds **1–8** revealed that all compounds (except compound **7**) were both geometrically pure and were configured *trans,* as derived from coupling constant *J*: 15.4–15.7 Hz for vinyl protons, observed at 7.60–8.09 ppm. The compound **7** was configured *cis* with coupling constant *J*: 10.4 Hz for vinyl protons. The aromatic ring protons were observed at the area of 7.0–8.0 ppm, as expected. The ^13 ^C-NMR spectra of all compounds, carbons of carbonyl groups were appeared about 187 ppm, as expected. HRMS results were also confirmed the chemical structures of the compounds.

**Figure 1.**
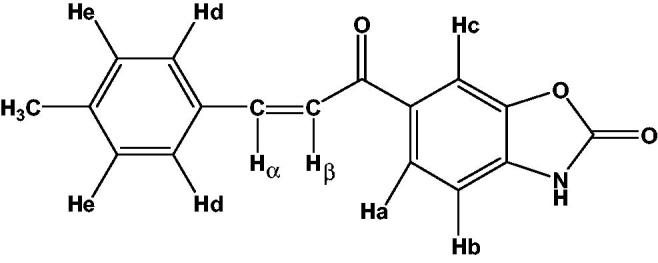
The details of ^1^H-NMR spectra of compound **2** as represantative ^1^H-NMR. δ (ppm) H_a_: 8.05 (dd, 1H, J_Ha-Hb_: 8.1 Hz, J_Ha-Hc_: 1.5 Hz), H_b_: 7.22 (d, 1H, J_Ha-Hb_: 8.1 Hz), H_c_: 8.08 (d, 1H, J_Ha-Hc_: 1.5 Hz), H_d_: 7.79 (d, 2H, J_Hd-He_: 8.0 Hz), H_e_: 7.27 (d, 2H, J_Hd-He_: 8.0 Hz), H_α_: 7.93 (d, 1H, J_Hα-Hβ_: 15.5 Hz), H_β_: 7.70 (d, 1H, J_Hα-Hβ_: 15.5 Hz) CH_3_ protons: 2.35 (s, 3H).

Although all the spectral results were presented in the experimental section, the details of ^1^H-NMR spectra of compound **2** are given in Figure 1 as an example.

### Cytotoxic/anticancer activity

The cytotoxicities of the synthesised compounds, **1–8**, have been investigated *in vitro* against oral squamous cancer cell line (HSC-2) and human normal oral cells (HGF and HPLF). The reference compounds used were DXR and 5-fluorouracil (5-FU) which are clinically in use for cancer treatment. Cytotoxicity results of compounds **1–8** were presented in Table 1. All of the compounds showed lower cytotoxicity than DXR but showed higher cytotoxicity than 5-FU. Cytotoxicities of the compounds were in the range of 4.0– 30.2 µM towards HSC-2 cell line. The compounds showed 1.3–9.4 times more cytotoxic than 5-FU. When the cytotoxicities of the compounds were considered, compound **4**, 6-[3–(4-Trifluoromethylphenyl)-2-propenoyl]-*3H*-benzoxazol-2-one, was found as the most potent cytotoxic molecule towards HSC-2 cell line.

**Table 1.** Cytotoxic activities of the compounds **1–8** towards human OSCC cell lines and human oral normal cells

	CC_50_(μM)								
	OSCC		Human normal oral cells						
	HSC-2	SD	HGF	SD	HPLF	SD	Mean	TS	PSE
Compounds	(A)						(B)	(B/A)	(B/A2)×100
**1**	22.1	1.2	71.0	9.5	83.3	14.0	77.2	3.5	16
**2**	10.1	0.4	87.7	18.5	105.3	3.1	96.5	9.5	94
**3**	14.5	2.9	88.3	17.9	84.0	0.0	86.2	5.9	41
**4**	4.0	0.6	24.7	0.6	26.3	0.5	25.5	6.4	159
**5**	6.9	0.5	55.4	19.7	50.3	3.5	52.9	7.7	111
**6**	10.1	0.5	53.0	5.3	76.0	7.0	64.5	6.4	64
**7**	30.2	3.2	76.3	9.3	70.0	2.0	73.2	2.4	8
**8**	11.9	0.2	98.0	50.2	32.7	1.5	65.3	5.5	46
5-FU*	37.7	0.0	>1000	0.0	>1000	0.0	>1000	>28.3	80.7
DXR*	0.5	0.1	0.7	0.0	>10	0.0	>5.3	>10.4	>2030

* 5-Fluorouracil (5-FU) and Doxorubicin (DXR) were used as reference drugs.

SD: standard deviation.

The first point to be considered for the compounds is whether they are tumour cytotoxins. Therefore, the compounds in the series were also evaluated against HGF and HPLF non-malignant cells and the data are presented in Table 1. Under clinical conditions, tumours are surrounded by different types of normal cells. Therefore, tumour selectivity values (TS) were calculated. TS were calculated for the compounds by dividing the average CC_50_ value towards normal cells into the average CC_50_ value towards cancer cell lines (Table 1). According to Table 1, the TS values of the compounds were greater than 1. This indicated that the compounds were tumour-selective. The cytotoxicity results pointed out that compound **2** had the highest TS value in the series.

Lead compounds should have both prominent cytotoxic potential and selective cytotoxicity for tumours. In order to identify the most promising compounds in terms of both good potencies and selectively cytotoxic, the PSE values were calculated according to equation i.e. [Average TS figure (determination of TS)/Average CC_50_ value (a measure of potency)^2^] × 100. PSE values were presented in Table 1. The PSE values of the compounds were in the range of 8–159. The number of compounds in the series with a PSE value greater than 30 is six. These six compounds appear to serve as lead compounds to improve new analogues.

The substituent on the phenyl ring rather than hydrogen was found to be a generally useful modification in increasing cytotoxicity (1.5 and 5.5 times) and selectivity (1.6–2.7 times). An exception was compound **7** for cytotoxicity and selectivity. This may result from the changes in the electronic structure of the phenyl ring. When methyl substituent is introduced into the 4 position of the phenyl ring (compound **2**), the cytotoxicity increased 2.2 times comparing to the non-substituted derivative (compound **1**), and TS increased 2.7 times. On the other hand, when the isopropyl group was substituted at the 4 position of phenyl ring in compound **6**, the increase in cytotoxicity (2.2 times) was the same as the methyl derivative (compound **2**) and the increase in selectivity of compound **6** was 1.8 times. These results suggested that the substitution of an alkyl group at the 4 position of phenyl was a useful modification in both the cytotoxicity and selectivity, especially when small size alkyl group substitution had better in increasing selectivity. It suggested that 4 position of the phenyl ring may play a crucial role in the interaction of the molecule with the active site of enzyme or protein. Compound **4**, wherein the trifluoromethyl group was substituted at the 4 position of the phenyl ring, supported this suggestion although the increase in selectivity in compound **4** was slightly less (1.8 times) than compound **2**. Additionally to TS, cytotoxicity increased dramatically (5.5 times) in compound **4**. This is noteworthy. When the electron-donating groups with resonance effect as 4-methoxy and 4-benzyloxy groups (compounds **3** and **8**) were substituted at the 4 position of the phenyl ring, the increase in cytotoxicity (1.5 and 1.9 times, respectively) was lower than compounds **2** and **4** which carry 4-methyl and 4-trifluoromethyl substituents on the phenyl ring. However, when the substitution of 4 position of phenyl was dimethylamine (compound **7**) cytotoxicity reduced 1.4 times comparing to the non-substituted derivative, compound **1**. In addition, substituents at 4 position in compounds **3** and **8** lead to a lower increase in selectivity comparing to the compounds **2** and **4**’s.

When the PSE values of the compounds were considered for the compounds having substitution at 4 position of phenyl ring, the results observed were interesting. The total electronic contributions of the substituents at 4 position of phenyl ring were as follows according to literature^61^; σ_isopropyl_: −0.15, σ_methyl_: −0.17, σ_methoxy_: −0.27, σ_dimethylamine_: −0.83 and σ_trifluoromethyl_: 0.54. The compounds bearing the substituent at issue were **6, 2, 3, 7,** and **4**. The results presented at Table 1 show that the replacement of hydrogen by electron-withdrawing substituent at the 4 position of the phenyl ring increased the PSE values of the compounds (except for compound **2**). It means as long as the Hammet value increased, the PSE value of the compound increased. There was a high positive correlation between σ and PSE values (r: 0.7918). It also reflects that electron-withdrawing substituents at the 4 position of phenyl had a positive effect on PSE.

Compound **4** carrying 4-trifluoromethyl substituent on phenyl ring was the most impressive compound of our design in terms of cytotoxicity and PSE value. This compound can be considered as a drug candidate for further investigations. The cytotoxicity and PSE value of the compound **4** were 5.5 and 9.9 times more potent than non-substituted compound **1**, respectively. While the cytotoxicity of compound **4** was 8 times low cytotoxic than DXR, it was 9.4 times more cytotoxic than reference compound 5-FU and its PSE (about 2 times) value was higher than 5-FU.

Another issue that can contribute to cytotoxicity can be the solubility of the compounds which was reflected by Partition Coefficients (logP). The logP values of the compounds were calculated by ChemDraw program (Ultra 7.0)[logP values: 2.71 (compound **1**), 3.20 (compound **2**), 2.58 (compound **3**), 3.63 (compound **4**), 2.32 (compound **5**), 3.94 (compound **6**), 2.99 (compound **7**), 4.31 (compound **8**)]. The correlations between logP and CC_50_ values were investigated and it was noticed that there was a low negative correlation (r: −0.2614).

### Carbonic anhydrase inhibitory effects

The human CA I and II inhibitory effects of the compounds **1–8** were reported for the first time in this study and the inhibition data are shown in Table 2. Acetazolamide (AZA) was used as a reference drug for both hCA I and II isoenzymes. The compounds **1–8** showed lower CA inhibitory effects than the reference drug, AZA. According to Table 2, IC_50_ values of the compounds **1–8** towards hCA I were in the range of 29.74–69.57 µM, while they were in the range of 18.14–48.46 µM towards hCA II isoenzyme. IC_50_ values of AZA were 16.58 and 8.37 µM towards hCA I and hCA II, respectively. According to the IC_50_ values, compound **4**, wherein the trifluoromethyl group was substituted at the 4 position of the phenyl ring showed the best activity (29.74 µM) towards hCA I and compound **8** carrying 4-benzyloxy substituent on phenyl ring showed the best activity (18.14 µM) towards hCA II.

**Table 2.** Inhibitory effects of the compounds **1–8** on hCA I and hCA II isoenzymes.

	IC_50_ (µM)			K*i* (µM)		
Compounds	hCA I	*r^2^*	hCA II	*r^2^*	hCA I	hCA II
**1**	58.72	0.9613	40.53	0.9392	70.58 ± 6.67	36.16 ± 13.35
**2**	33.00	0.9546	45.59	0.9786	38.80 ± 1.93	32.12 ± 6.60
**3**	47.46	0.9406	48.13	0.9854	30.49 ± 11.26	36.20 ± 10.26
**4**	29.74	0.9397	45.0	0.9778	30.75 ± 3.49	19.2 ± 2.19
**5**	69.57	0.9375	48.46	0.9509	63.04 ± 15.45	35.20 ± 8.36
**6**	52.11	0.9496	30.13	0.9356	37.05 ± 7.77	33.29 ± 6.15
**7**	40.29	0.9533	29.74	0.9538	52.25 ± 9.97	10.85 ± 2.14
**8**	57.75	0.9509	18.14	0.9397	28.37 ± 6.63	37.96 ± 2.36
AZA*	16.58	0.9887	8.37	0.9825	30.18 ± 7.77	4.41 ± 0.55

* Acetazolamide (AZA) was used as a standard inhibitor for both hCA I and II isoenzymes. *r*^2^: is a statistical measure of how close the data are to the fitted regression line. It is also known as the coefficient of determination, or the coefficient of multiple determinations for multiple regressions^59^.

According to Table 2, K*_i_* values (inhibitory potency) of the compounds **1–8** towards hCA I were in the range of 28.37 ± 6.63 – 70.58 ± 6.67 µM towards hCA I isoenzyme and they were in the range of 10.85 ± 2.14 – 37.96 ± 2.36 µM towards hCA II isoenzyme. K_i_ values of AZA were 30.18 ± 7.77 µM and 4.41 ± 0.55 µM towards hCA I and hCA II, respectively. So, some compounds (compounds **3, 4, 6,** and **8**) had similar K*_i_* values with AZA towards hCA I while all compounds had higher K*_i_* values than AZA towards hCA II which suggests that they are worse inhibitor than AZA.

## Conclusions

Newly synthesized 6-(3-aryl-2-propenoyl)-2(*3H*)-benzoxazolones, the compounds **1–8**, were reported here for the first time with their cytotoxic properties and potential inhibitory effects on hCA I and II. According to the cytotoxicity results of the compounds, compound **4** was the most impressive lead compound of the study with remarkably PSE value (159) for further testing and investigations. On the other hand, according to K*_i_* values compounds **2, 3, 4, 6,** and **8** can be considered for the development of new CA I inhibitors due to similar K_i_ values to AZA but they are not suitable derivatives for the development of new CA II inhibitors since they had higher K*_i_* values than AZA.
